# Trends in Antimicrobial Consumption in Pakistan (2016‐2028): Retrospective Observational Study With Forecasting

**DOI:** 10.2196/81288

**Published:** 2026-04-28

**Authors:** Zikria Saleem, Brian Godman, Zunaira Akbar, Abdul Haseeb, Muhammad Usman Qamar, Anees ur Rehman, Mahmoud E Elrggal

**Affiliations:** 1 Department of Pharmacy Practice, College of Pharmacy, Qassim University, Buraydah, Saudi Arabia, 966 504824750; 2 Department of Public Health Pharmacy and Management, School of Pharmacy, Sefako Makgatho Health Sciences University, Ga-Rankuwa, South Africa; 3 Department of Pharmacy Practice, Riphah Institute of Pharmaceutical Sciences, Riphah International University, Lahore, Pakistan; 4 Department of Pharmacy Practice, Faculty of Pharmacy, University of Tabuk, Tabuk, Saudi Arabia; 5 Institute of Microbiology, Faculty of Life Sciences, Government College University, Faisalabad, Pakistan; 6 Department of Pharmacy Practice, Faculty of Pharmacy, Bahauddin Zakariya University, Multan, Pakistan; 7 College of Medicine, Al-Qunfudah, Umm Al-Qura University, Makkah, Saudi Arabia

**Keywords:** trends, projections, antimicrobial consumption, Pakistan, implications, antimicrobial stewardship, resistance

## Abstract

**Background:**

Antimicrobial resistance is a public health crisis exacerbated by the irrational use of antibiotics, particularly in low- and middle-income countries. Pakistan, one of the highest consumers of antibiotics globally, faces unique challenges, including unregulated sales, overuse of broad-spectrum antibiotics, and inadequate stewardship programs.

**Objective:**

This study aimed to analyze antibiotic consumption trends in Pakistan from 2016 to 2023, project future use through 2028, and evaluate the subsequent implications for antimicrobial resistance and antimicrobial stewardship programs.

**Methods:**

Antibiotic sales data were retrieved for Pakistan from the IQVIA MIDAS database spanning 2016 to 2023. Data were converted to defined daily doses (DDDs) and DDD per 1000 inhabitants per day (DID) using the World Health Organization Anatomical Therapeutic Chemical classification system. Data cleaning, statistical analyses, and data visualization were performed using R software (version 4.3.2) and Microsoft Excel. Trends were analyzed using linear regression, while future projections (2024‐2028) were developed using trend-based models. Descriptive analysis was performed, and visualizations were used to illustrate findings.

**Results:**

The total antibiotic consumption in Pakistan from 2016 to 2023 was 12.88 billion DDDs. Broad-spectrum penicillins and fluoroquinolones, each accounting for 37.7 DID, were the most consumed classes. The analysis revealed significant increases in the consumption of macrolides (+76%; rising from 2.26 to 3.99 DID) and cephalosporins (+36%; from 2.87 to 3.89 DID) from 2016 to 2023, with macrolides projected to reach 5.79 DID by 2028. Reserve antibiotics, including oxazolidinones (+354%; from 0.03-to 0.014 DID) and glycylcycline (+236%; from 0.001 to 0.0003 DID), also showed appreciable increases, reflecting greater reliance on last-line therapies. In contrast, aminoglycosides (−36%; from 0.013 to 0.14 DID) and narrow-spectrum penicillins (−30%; from 0.008 to 0.005 DID) experienced notable declines.

**Conclusions:**

The study highlights a concerning overreliance on broad-spectrum and reserve antibiotics in Pakistan, thus underscoring the urgent need for robust antimicrobial stewardship programs and stricter regulation of over-the-counter antibiotic sales to rationalize antibiotic use. Future efforts should focus on addressing gaps in prescribing practices, improving diagnostic capacity, and monitoring stewardship program outcomes to mitigate resistance development and preserve antibiotic efficacy.

## Introduction

Antibiotics have revolutionized modern medicine, playing a critical role in preventing and treating bacterial infections [1]. However, their widespread and often irrational use has led to an appreciable increase in antimicrobial resistance (AMR) in recent years [2]. AMR is one of the greatest public health challenges of the 21st century, threatening the effectiveness of antibiotics and the ability to treat common infections, ultimately increasing morbidity, mortality, and health care costs [2-4]. In low- and middle-income countries (LMICs), including Pakistan, where health care systems are underresourced and antibiotic use is often unregulated, this issue becomes even more pronounced [5-8].

Pakistan is among the top consumers of antibiotics globally, a distinction that underscores the urgent need for comprehensive surveillance and targeted interventions to address current concerns with their overuse [9]. Key factors driving high antibiotic consumption in the country include easy availability of over-the-counter antibiotics, an insufficient health care infrastructure, lack of awareness among prescribers and the public regarding antibiotics and AMR, and sociocultural practices that promote self-medication [5,6,10]. The unregulated sale of antibiotics and the proliferation of generic brands further exacerbate the problem, leading to overuse and misuse of broad-spectrum antibiotics including those from the World Health Organization (WHO) Watch List [7,11-13]. The COVID-19 pandemic has further complicated the landscape of antibiotic use [14-16]. There was an unprecedented surge in the use of antibiotics during the early stages of the pandemic, driven by the misperception that antibiotics could prevent or treat COVID-19 [16-18]. These challenges, compounded by inadequate national stewardship policies and concerns with limited knowledge and antimicrobial stewardship activities, have contributed to a significant rise in AMR in Pakistan, posing a major threat to public health and economic stability [10,19].

Surveillance of antibiotic consumption is a cornerstone in combating AMR, providing insights into use patterns and identifying areas for intervention [9,20]. The number of defined daily doses (DDDs) per 1000 inhabitants per day (DID) is a standardized metric recommended by the WHO for measuring antibiotic consumption. This metric allows for meaningful comparisons over time and across countries [21]. Understanding trends in antibiotic consumption, particularly in the context of Pakistan, is crucial for informing national policies, optimizing antimicrobial stewardship programs (ASPs), and aligning with the global action plan on AMR [5]. Assessing antibiotic use patterns across sectors has gained importance following recent deliberations of the United Nations General Assembly (UNGA) on AMR, which emphasize increasing the use of antibiotics from the WHO Access List to help reduce AMR [22]. This is important in Pakistan, along with other LMICs, where we see high rates of use of antibiotics from the Watch and Reserve lists with their greater resistance potential [9,12]. Such trends must be reversed if Pakistan is to meet its National Action Plan targets and the UNGA goal of achieving 70% of antibiotic use from Access List antibiotics [22]. Previous studies have highlighted high consumption rates of antibiotics, including cephalosporins, fluoroquinolones, and macrolides, in Pakistan; however, there is currently a lack of comprehensive analyses regarding long-term trends and projections for antibiotic use in Pakistan [9,17]. This gap in knowledge hinders the country’s ability to meet its National Action Plan on AMR, which is already a challenge, and contribute meaningfully to global efforts in reducing antibiotic misuse [5]. This study provides a detailed analysis of antibiotic consumption trends in Pakistan from 2016 to 2023, with projections through 2028. By analyzing consumption data and forecasting across 13 major antibiotic classes, this study establishes a counterfactual baseline of expected use and aims to highlight secular trends; identify statistically significant changes; and assess the impact of potential interventions, such as the COVID-19 pandemic, on use patterns. The findings are intended to inform policymakers, health care providers, and researchers about the current state of antibiotic use in Pakistan and the critical need for targeted stewardship efforts.

## Methods

### Data Source and Collection

Antibiotic consumption data for Pakistan were retrieved from the comprehensive IQVIA MIDAS database, which consolidates pharmaceutical sales data, as reported in a recent study by Klein et al [9]. The data covered the period from 2016 to 2023 and included retail pharmacy sales, capturing estimated use trends for antibiotics across the country. The sales data, recorded in estimated grams of active ingredients, were systematically converted into DDD using the Anatomical Therapeutic Chemical classification system and methodologies recommended by the WHO Collaborating Centre for Drug Statistics Methodology. The DDD conversion accounted for variations in the route of administration (oral and parenteral) for each molecule.

### Data Processing

Antibiotic molecules were classified according to the Anatomical Therapeutic Chemical or DDD Index 2026 [23]. For combination drugs, components were broken into their individual active molecules, and consumption was attributed to the main antibiotic molecule based on its 1 daily dose. Antibiotic consumption rates were calculated as DID, enabling cross-year and cross-population comparison. Population data for Pakistan were sourced from the World Bank and national government statistics.

### Study Period and Variables

The study analyzed 13 antibiotic classes, including macrolides, cephalosporins, fluoroquinolones, carbapenems, tetracyclines, and others. Variables included annual DIDs for each antibiotic class, percentage changes from 2016 to 2023, and projections (2024‐2028).

### Statistical Analysis

Antibiotic classes were categorized as increasing, decreasing, or stable based on percentage changes in DIDs. Classes with changes >10% were considered increasing, <−10% as decreasing, and between −10% to 10% as stable. This operational threshold was adopted by the authors as a pragmatic balance between clinical relevance and statistical sensitivity. Data cleaning, statistical analyses, and data visualization were performed using R software (version 4.3.2; R Foundation for Statistical Computing; Posit) and Microsoft Excel. For each antibiotic class, linear regression models were applied to evaluate trends from 2016 to 2023. Slope coefficients indicated the rate of annual change. Future antibiotic use (2024‐2028) was projected using the coefficients from linear regression models. The projections assumed a consistent linear trend in use, with results expressed in DIDs. A +10% or −10% SE margin was incorporated to reflect potential variability due to external factors such as policy changes and AMR management programs. Line graphs with distinct markers were used to show historical trends and projections. Bar charts were used to compare percentage changes, emphasizing significant trends.

### Ethical Considerations

Ethics approval for the study was obtained from the Research Ethics Committee of the Department of Pharmacy Practice, Faculty of Pharmacy, Bahauddin Zakariya University, Multan (BZU-FOPDPP-2456). Participant consent was not required as the study was based on aggregated sales data and did not involve human participants or identifiable personal information. All data were aggregated and anonymized, ensuring compliance with ethical standards for research.

## Results

The analysis of antibiotic use in Pakistan between 2016 and 2023, alongside projections for 2024 to 2028, revealed important trends and insights. Table 1 and Table S1 in Multimedia Appendix 1 highlight the annual consumption of antibiotics as DDDs and DID. The total antibiotic consumption in Pakistan during the study period from 2016 to 2023 was measured at an estimated 12.88 billion DDDs. Among the antibiotic classes, broad-spectrum penicillins and fluoroquinolones were the most consumed, contributing significantly to the total DDDs. These findings emphasize the dominant role of these classes in driving overall antibiotic use, highlighting their critical impact on AMR trends. Over the study period, macrolides exhibited the highest growth (+76%), increasing from 2.26 to 3.98 DID, and are projected to reach 5.79 DID by 2028. Similarly, cephalosporins increased by 35.8% from 2.86 to 3.89 DID, with projections suggesting further growth to 4.76 DID. Conversely, tetracyclines showed a slight decline (−3.3%) from 2.19 to 2.12 DID, with projections indicating a continued decrease to 2.00 DID by 2028.

**Table 1.** Consumption pattern of antibiotics as defined daily dose (DDD) per 1000 inhabitants per day (DID) in Pakistan.

Antibiotic class	Year-wise DDD per 1000 DID							
	2016	2017	2018	2019	2020	2021	2022	2023
Broad-spectrum penicillins	4.8475	4.5654	4.6502	4.7367	4.5420	4.6185	4.9834	4.7300
Fluoroquinolones	4.5628	4.4010	4.6072	4.5863	4.3664	4.8303	5.2147	5.1958
Cephalosporins	2.8666	2.8807	3.1315	3.2945	3.1519	3.6104	3.9696	3.8936
Macrolides	2.2590	2.2778	2.5770	2.6436	3.3594	3.9336	4.1285	3.9864
Tetracyclines	2.1930	2.2175	2.2884	2.2098	2.1028	2.1524	2.0863	2.1199
Sulfonamides	1.2224	1.0970	1.1353	1.2119	1.1897	1.0901	1.0633	1.0125
Aminoglycosides	0.1324	0.1164	0.1049	0.0849	0.0797	0.0876	0.0992	0.0851
Oxazolidinones	0.0313	0.0428	0.0555	0.0632	0.0707	0.1026	0.1209	0.1420
Narrow-spectrum penicillins	0.0084	0.0069	0.0098	0.0087	0.0069	0.0086	0.0083	0.0059
Carbapenems	0.0071	0.0076	0.0094	0.0101	0.0106	0.0207	0.0210	0.0218
Glycopeptide	0.0015	0.0014	0.0018	0.0021	0.0025	0.0038	0.0034	0.0045
Glycylcycline	0.0001	0.0001	0.0001	0.0002	0.0001	0.0002	0.0002	0.0003
Other antibacterials	0.0460	0.0451	0.0453	0.0470	0.0448	0.0469	0.0441	0.0377

**Figure 1.**
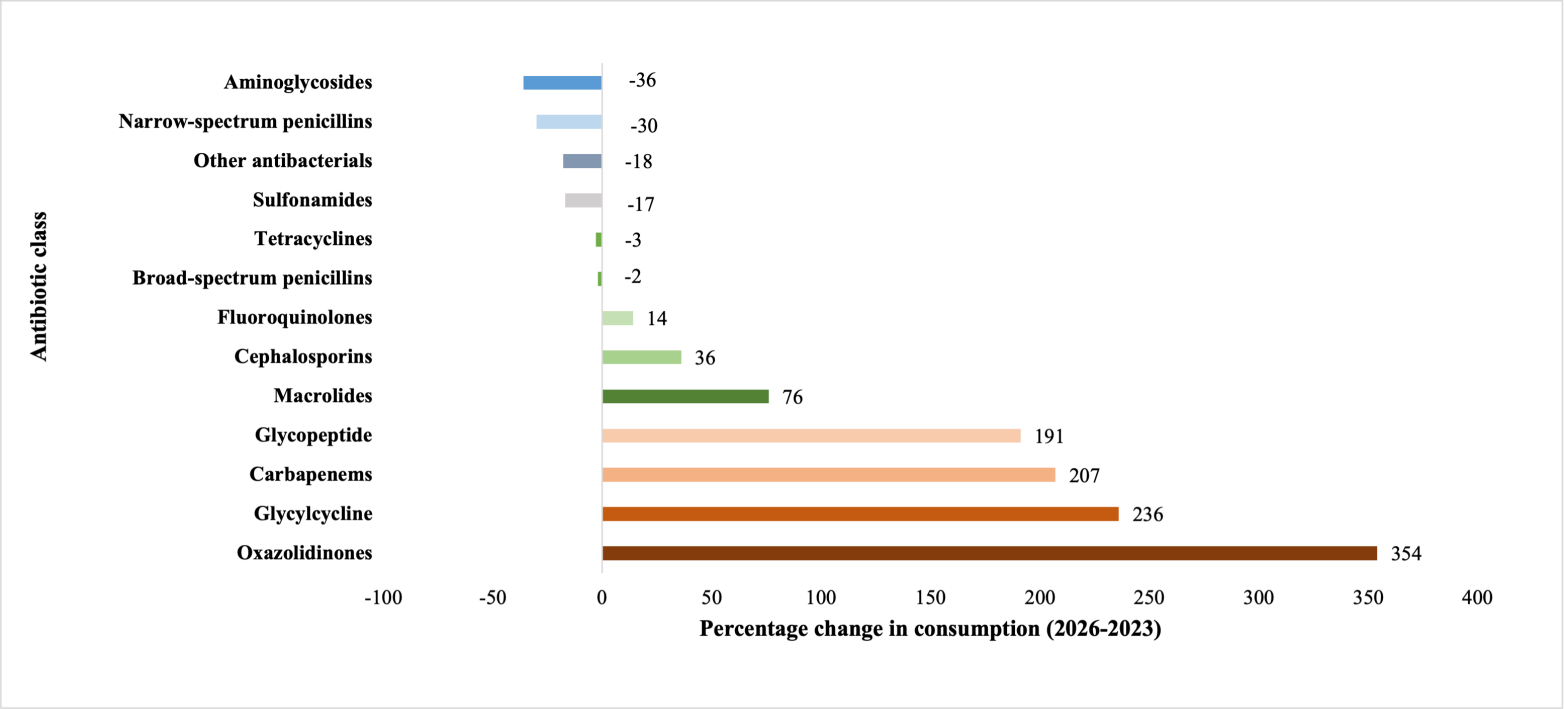
Cluster changes in consumption between 2016 and 2023.

Table 2 presents year-on-year percentage changes. Significant increases were observed for carbapenems (+207%), glycopeptides (+191%), and glycylcycline (+236%), driven largely by higher demand in clinical settings. Oxazolidinones exhibited the most dramatic increase (+354%), reflecting increased reliance on reserve antibiotics. In contrast, aminoglycosides (−36%), narrow-spectrum penicillins (−30%), and sulfonamides (−17%) experienced a notable decline, possibly due to shifts in prescribing preferences. Figure 1 illustrates trends in antibiotic use, with aminoglycosides showing a significant reduction and oxazolidinones exhibiting the largest increase in consumption over the previous 8 years (2016‐2023).

**Table 2.** Percentage change in consumption.

Antibiotic class	Year-wise change in consumption (%)							
	(2016‐2017)	(2017‐2018)	(2018‐2019)	(2019‐2020)	(2020‐2021)	(2021‐2022)	(2022‐2023)	(2016‐2023)
Broad-spectrum penicillins	−6	2	2	−4	2	8	−5	−2
Fluoroquinolones	−4	5	0	−5	11	8	0	14
Cephalosporins	0	9	5	−4	15	10	−2	36
Macrolides	1	13	3	27	17	5	−3	76
Tetracyclines	1	3	-3	−5	2	−3	2	−3
Sulfonamides	−10	3	7	−2	-8	−2	−5	−17
Aminoglycosides	−12	−10	−19	−6	10	13	−14	−36
Oxazolidinones	37	30	14	12	45	18	18	354
Narrow-spectrum penicillins	−18	41	−11	−21	26	−3	−29	−30
Carbapenems	7	24	7	5	95	1	4	207
Glycopeptide	−12	30	23	17	52	−10	30	191
Glycylcycline	−10	2	96	−9	60	10	17	236
Other antibacterials	−2	0	4	−5	5	−6	−15	−18

**Figure 2.**
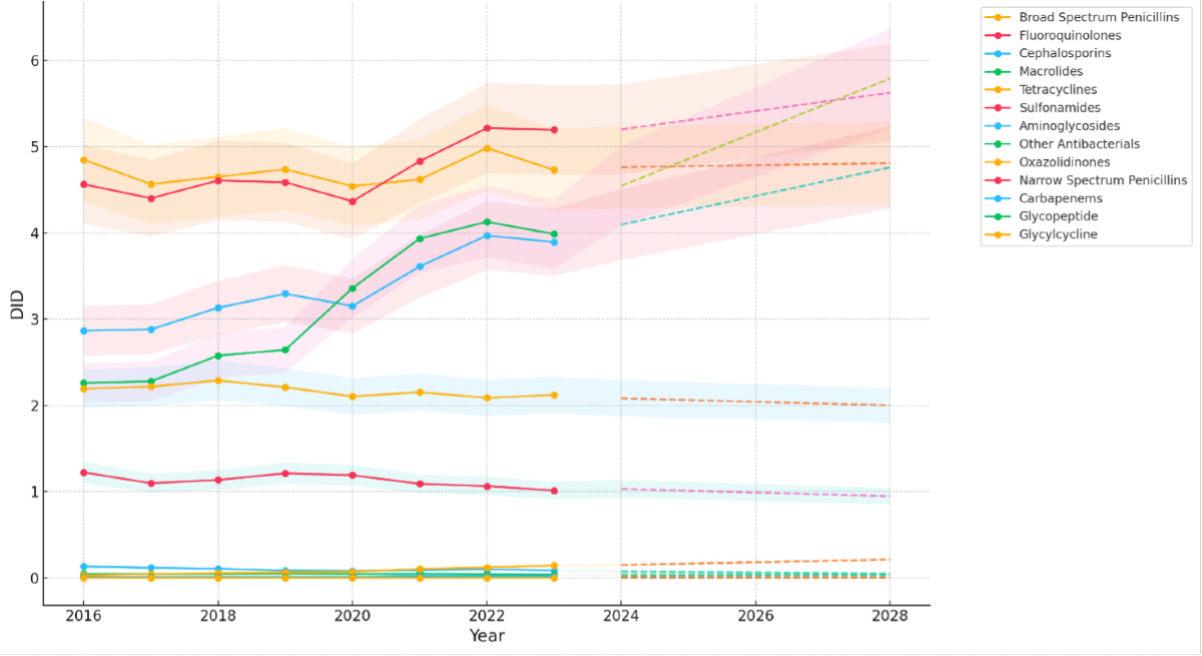
Trends and projections of antibiotic use in Pakistan (2016‐2028). DID: defined daily dose per 1000 inhabitants per day.

Figure 2 demonstrates historical trends and future projections until 2028, with projections indicated by dotted lines. Broad-spectrum antibiotics such as macrolides and cephalosporins show steep upward trajectories (2.25 DID to 5.78 DID and 2.86 DID to 4.75 DID, respectively), signaling the potential overuse of these agents, whereas fluoroquinolones, a critical group, are projected to increase modestly to 5.63 DID by 2028. The inclusion of a +10% or −10% margin of error reflects variability due to external factors such as policy changes or antimicrobial stewardship interventions.

## Discussion

### Overview

This study analyzed antibiotic consumption trends in Pakistan from 2016 to 2023, with projections through 2028, highlighting critical insights into patterns of use and their implications for AMR and ASP. The findings align with global trends but also underscore the unique challenges faced by LMICs, such as Pakistan, in managing antibiotic consumption [24]. The total antibiotic consumption in Pakistan was measured at 12.88 billion DDDs, reflecting a 17% increase since 2016. This trend parallels global findings, where antibiotic consumption rose by 20.9% during a similar period, although the growth rate was slower compared to the 35.5% increase observed from 2008 to 2015 [25]. However, the COVID-19 pandemic significantly impacted global antibiotic use, leading to temporary reductions, particularly in high-income countries, where outpatient antibiotic use is dominant [9,26-28]. In Pakistan, as in other LMICs, a rebound was observed postpandemic, with consumption exceeding prepandemic levels by 2023, driven by unregulated access and increased reliance on broad-spectrum antibiotics [28,29].

Broad-spectrum penicillins and fluoroquinolones were the most consumed antibiotic classes in Pakistan, accounting for 37.7 DID each in 2023. This heavy reliance on broad-spectrum antibiotics is consistent with trends observed in other LMICs such as India and Bangladesh, where similar patterns have been linked to limited diagnostic capacity and the widespread availability of over-the-counter antibiotics [9,30,31]. In contrast, reserve antibiotics, including carbapenems, oxazolidinones, and glycylcycline, showed dramatic increases in use. These trends highlight the growing demand for last-line antibiotics in critical care settings, reflecting rising resistance to first-line therapies [13,32]. Several factors contribute to the accelerated rise in antibiotic use in developing countries, including environmental, socioeconomic, and cultural determinants, with economic growth appearing to be the most influential driver in lower-income settings [9]. During the COVID-19 pandemic, antibiotic sales also increased considerably due to empirical use in the setting of diagnostic uncertainty and concerns about bacterial coinfections [33]. The projected escalation in the use of macrolides and cephalosporins (2.25 DID to 5.78 DID and 2.86 DID to 4.75 DID, respectively) reflects an increasing dependence on broad-spectrum antibiotics. This growing dependence on Watch group antibiotics deviates from the WHO’s recommended target of maintaining at least 60% of total consumption within the Access category, potentially accelerating the selection of multidrug-resistant pathogens [33].

Economic growth, urbanization, and health care infrastructure gaps are significant drivers of antibiotic overuse in Pakistan. As seen in other middle-income countries, rising gross domestic product and rapid urbanization often correlate with increased antibiotic consumption [34]. However, this growth comes at the expense of proper regulatory oversight, leading to widespread misuse. In Pakistan, the lack of robust ASPs and weak enforcement of prescription-only antibiotic policies exacerbate the problem [6,12,35]. The WHO’s Access, Watch, and Reserve (AWaRe) classification system has been pivotal in promoting rational antibiotic use globally [36]. However, in Pakistan, as in many LMICs, the Watch and Reserve categories dominate consumption, reflecting poor alignment with the AWaRe recommendations [25]. In contrast, high-income countries have successfully reduced the use of high-risk antibiotics such as fluoroquinolones through stringent regulatory warnings and public awareness campaigns [9,37]. The rising consumption of fluoroquinolones and reserve antibiotics in Pakistan highlights the urgent need for similar regulatory interventions [17]. This study underscores the critical need for comprehensive ASPs tailored to the local context [38]. Strengthening regulatory frameworks, enhancing diagnostic capacity, and promoting public awareness are essential to curbing antibiotic misuse [39,40]. Investments in preventive measures, such as improved water, sanitation, and hygiene infrastructure, along with robust vaccination programs, could also significantly reduce the burden of infectious diseases and, consequently, the need for antibiotics in Pakistan [41,42].

### Principal Findings

The major findings of the study are as follows:

- Total antibiotic consumption in Pakistan (2016‐2023) reached 12.88 billion DDDs, with broad-spectrum penicillins and fluoroquinolones accounting for nearly half.
- Annual consumption of macrolides (+76%) and cephalosporins (+36%) increased sharply, with macrolides projected to reach 5.79 DID by 2028.
- Reserve antibiotics, including oxazolidinones and glycylcycline, showed marked growth, reflecting increased use of last-line therapies, while aminoglycosides and narrow-spectrum penicillins declined.

### Study Limitations and Future Research

While the data in this report provide valuable insights, they have inherent limitations. First, the IQVIA MIDAS data primarily capture retail pharmacy sales and do not include hospital procurement or inpatient use, which may lead to underrepresentation of antibiotics predominantly used in hospital settings (eg, carbapenems and glycopeptides) or for severe infections. No distinction was made between prescription-only and over-the-counter antibiotics, as consumption estimates were derived from aggregated sales data. Second, the dataset lacks indication-level, health care setting–level, and prescriber-level information, preventing analysis of the clinical context or appropriateness of antibiotic use. Third, the projections for 2024 to 2028 assume linear trends, which may not account for unforeseen policy changes, health care developments, or epidemics. The study does not address regional variations within Pakistan, which could provide additional insights into localized drivers of antibiotic use. Additionally, the study focused exclusively on human antibiotic consumption, excluding veterinary and agricultural use, which are critical components of a One Health approach to AMR. Future research should integrate these dimensions to provide a more comprehensive understanding of antibiotic use trends.

### Conclusions

The findings of this study highlight the urgent need for targeted interventions to optimize antibiotic use in Pakistan. The high reliance on broad-spectrum and reserve antibiotics, coupled with the rapid rebound in consumption after the pandemic, underscores the importance of strengthening ASPs and regulatory oversight. By aligning national policies with global frameworks following UNGA recommendations, Pakistan can mitigate the risks of AMR and ensure sustainable antibiotic use.

## Supplementary material

10.2196/81288 Multimedia Appendix 1 Year-wise consumption of antibiotics in terms of defined daily dose and projections in terms of defined daily dose per 1000 inhabitants per day.

## Abbreviations

AMR: antimicrobial resistance
ASP: antimicrobial stewardship program
AWaRe: Access, Watch, Reserve
DDD: defined daily dose
DID: defined daily dose per 1000 inhabitants per day
LMIC: low- and middle-income country
UNGA: United Nations General Assembly
WHO: World Health Organization
