# Supplementary material for: Trends in Antimicrobial Consumption in Pakistan (2016‐2028): Retrospective Observational Study With Forecasting
Source: JMIR Public Health Surveill. 2026 Apr 28;12:e81288. doi: 10.2196/81288 (PMC13124083; doi:10.2196/81288)
Supplement: Multimedia Appendix 1 [file publichealth-v12-e81288-s001.docx]

**Supplementary Data**


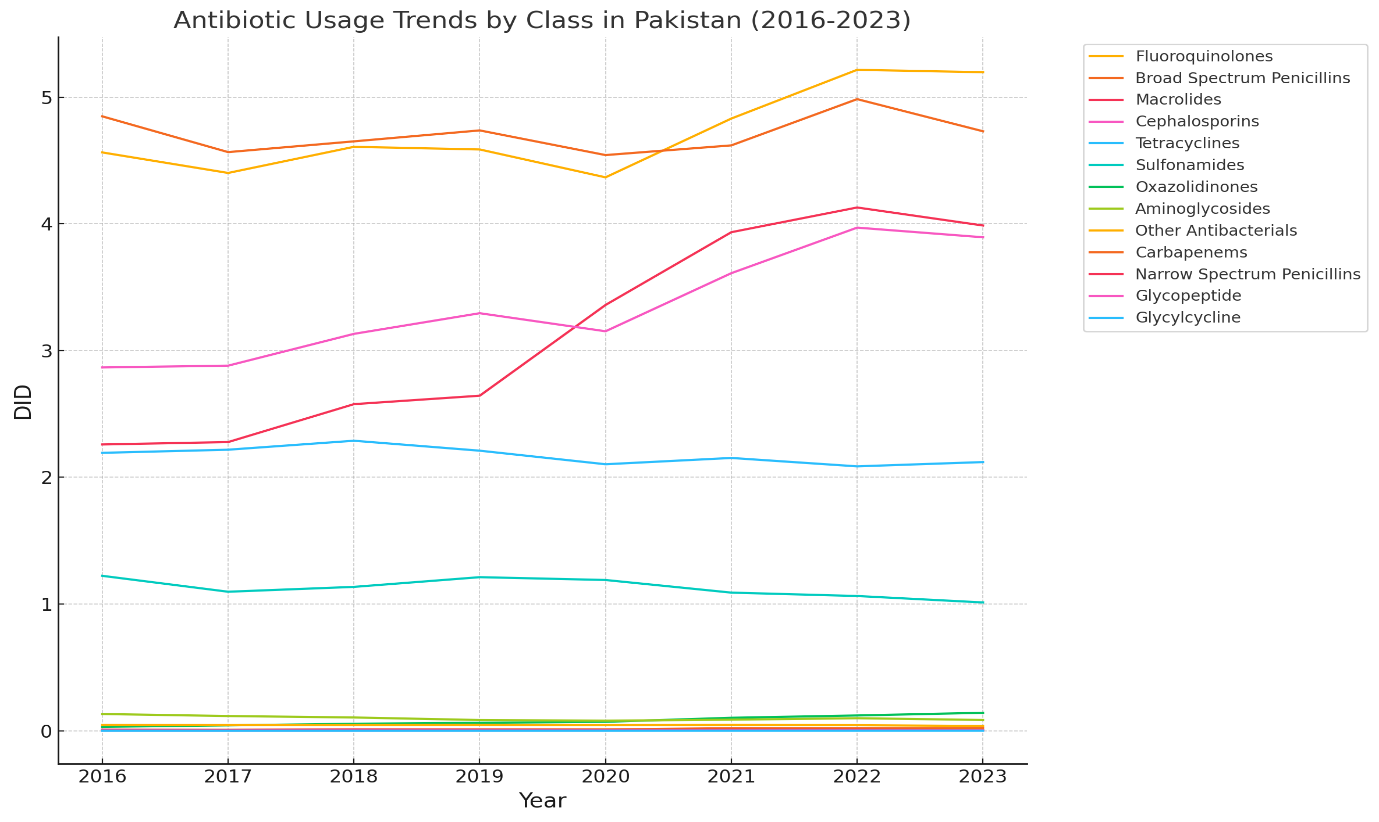


Figure S1: Antibiotic Usage Trend

Table S1: No of DDDs consumption

| Class | 2016 | 2017 | 2018 | 2019 | 2020 | 2021 | 2022 | 2023 | Total |
| --- | --- | --- | --- | --- | --- | --- | --- | --- | --- |
| TOTAL | 1,416,577,994 | 1,394,858,766 | 1,492,855,785 | 1,540,342,099 | 1,569,616,707 | 1,731,933,271 | 1,871,348,565 | 1,864,104,907 | 12,881,638,093 |
| Broad Spectrum Penicillins | 377,756,835 | 360,599,831 | 372,905,536 | 386,060,893 | 376,659,784 | 390,086,686 | 428,903,365 | 415,210,706 | 3,108,183,636 |
| Fluoroquinolones | 355,570,554 | 347,620,711 | 369,456,019 | 373,803,220 | 362,095,425 | 407,967,779 | 448,814,862 | 456,097,374 | 3,121,425,944 |
| Cephalosporins | 223,383,714 | 227,533,453 | 251,119,180 | 268,513,168 | 261,379,169 | 304,934,472 | 341,649,303 | 341,787,435 | 2,220,299,895 |
| Macrolides | 176,037,640 | 179,911,673 | 206,648,007 | 215,462,198 | 278,590,055 | 332,233,485 | 355,331,411 | 349,937,968 | 2,094,152,438 |
| Tetracyclines | 170,892,933 | 175,154,397 | 183,506,314 | 180,111,946 | 174,380,185 | 181,797,300 | 179,558,650 | 186,092,177 | 1,431,493,901 |
| Sulfonamides | 95,260,518 | 86,648,478 | 91,039,440 | 98,774,027 | 98,660,711 | 92,067,022 | 91,519,228 | 88,877,300 | 742,846,724 |
| Aminoglycosides | 10,314,604 | 9,191,473 | 8,412,886 | 6,916,682 | 6,611,196 | 7,396,677 | 8,539,192 | 7,471,566 | 64,854,275 |
| Other Antibacterials | 3,583,700 | 3,561,434 | 3,629,614 | 3,833,345 | 3,712,473 | 3,965,230 | 3,795,171 | 3,305,825 | 29,386,793 |
| Oxazolidinones | 2,440,696 | 3,378,065 | 4,452,877 | 5,149,553 | 5,860,521 | 8,667,182 | 10,401,608 | 12,468,523 | 52,819,025 |
| Narrow Spectrum Penicillins | 656,754 | 548,670 | 785,532 | 709,222 | 568,362 | 726,630 | 715,783 | 521,420 | 5,232,371 |
| Carbapenems | 553,759 | 597,851 | 753,765 | 820,514 | 878,407 | 1,748,932 | 1,803,324 | 1,916,850 | 9,073,402 |
| Glycopeptide | 119,648 | 106,692 | 140,372 | 174,891 | 208,854 | 323,041 | 295,643 | 392,664 | 1,761,805 |
| Glycylcycline | 6,639 | 6,038 | 6,242 | 12,442 | 11,564 | 18,834 | 21,025 | 25,100 | 107,884 |

Table S2: Trends and projections in terms of DID

| Year | 2016 | 2017 | 2018 | 2019 | 2020 | 2021 | 2022 | 2023 | 2024 | 2025 | 2026 | 2027 | 2028 |
| --- | --- | --- | --- | --- | --- | --- | --- | --- | --- | --- | --- | --- | --- |
| TOTAL | 18.1782 | 17.6596 | 18.6164 | 18.8989 | 18.9275 | 20.5057 | 21.7429 | 21.2355 | 22.0160 | 22.5817 | 23.1473 | 23.7130 | 24.2786 |
| Broad Spectrum Penicillins | 4.8475 | 4.5654 | 4.6502 | 4.7367 | 4.5420 | 4.6185 | 4.9834 | 4.7300 | 4.7616 | 4.7732 | 4.7848 | 4.7965 | 4.8081 |
| Fluoroquinolones | 4.5628 | 4.4010 | 4.6072 | 4.5863 | 4.3664 | 4.8303 | 5.2147 | 5.1958 | 5.1999 | 5.3064 | 5.4130 | 5.5195 | 5.6260 |
| Cephalosporins | 2.8666 | 2.8807 | 3.1315 | 3.2945 | 3.1519 | 3.6104 | 3.9696 | 3.8936 | 4.0959 | 4.2617 | 4.4275 | 4.5933 | 4.7592 |
| Macrolides | 2.2590 | 2.2778 | 2.5770 | 2.6436 | 3.3594 | 3.9336 | 4.1285 | 3.9864 | 4.5456 | 4.8567 | 5.1677 | 5.4788 | 5.7899 |
| Tetracyclines | 2.1930 | 2.2175 | 2.2884 | 2.2098 | 2.1028 | 2.1524 | 2.0863 | 2.1199 | 2.0811 | 2.0611 | 2.0411 | 2.0210 | 2.0010 |
| Sulfonamides | 1.2224 | 1.0970 | 1.1353 | 1.2119 | 1.1897 | 1.0901 | 1.0633 | 1.0125 | 1.0316 | 1.0102 | 0.9888 | 0.9674 | 0.9461 |
| Aminoglycosides | 0.1324 | 0.1164 | 0.1049 | 0.0849 | 0.0797 | 0.0876 | 0.0992 | 0.0851 | 0.0734 | 0.0678 | 0.0621 | 0.0565 | 0.0508 |
| Other Antibacterials | 0.0460 | 0.0451 | 0.0453 | 0.0470 | 0.0448 | 0.0469 | 0.0441 | 0.0377 | 0.0414 | 0.0406 | 0.0399 | 0.0392 | 0.0385 |
| Oxazolidinones | 0.0313 | 0.0428 | 0.0555 | 0.0632 | 0.0707 | 0.1026 | 0.1209 | 0.1420 | 0.1490 | 0.1647 | 0.1803 | 0.1960 | 0.2116 |
| Narrow Spectrum Penicillins | 0.0084 | 0.0069 | 0.0098 | 0.0087 | 0.0069 | 0.0086 | 0.0083 | 0.0059 | 0.0071 | 0.0069 | 0.0067 | 0.0065 | 0.0063 |
| Carbapenems | 0.0071 | 0.0076 | 0.0094 | 0.0101 | 0.0106 | 0.0207 | 0.0210 | 0.0218 | 0.0245 | 0.0269 | 0.0294 | 0.0318 | 0.0342 |
| Glycopeptide | 0.0015 | 0.0014 | 0.0018 | 0.0021 | 0.0025 | 0.0038 | 0.0034 | 0.0045 | 0.0046 | 0.0051 | 0.0055 | 0.0060 | 0.0064 |
| Glycylcycline | 0.0001 | 0.0001 | 0.0001 | 0.0002 | 0.0001 | 0.0002 | 0.0002 | 0.0003 | 0.0003 | 0.0003 | 0.0004 | 0.0004 | 0.0004 |
